# Is higher psychotropic medication burden associated with involuntary treatment under the Mental Health Act? A four-year Australian cohort study

**DOI:** 10.1186/s12888-020-02661-6

**Published:** 2020-06-11

**Authors:** Amanda J. Wheeler, Jie Hu, Caitlin Profitt, Sara S. McMillan, Theo Theodoros

**Affiliations:** 1 Menzies Health Institute Queensland, Nathan Campus, Griffith University, 170 Kessels Road, Brisbane, QLD 4111 Australia; 2 Faculty of Medical and Health Sciences, Auckland University, Auckland, New Zealand; 3 School of Pharmacy and Pharmacology, Griffith University, Brisbane, Australia; 4 University of Queensland Faculty of Medicine; Metro South Mental Health Services, Brisbane, Australia

**Keywords:** Psychotropics, Medication, Involuntary treatment, Antipsychotic, Polypharmacy

## Abstract

**Background:**

Involuntary treatment for individuals who lack sufficient capacity to make informed decisions regarding treatment has been associated with increased rates of injectable antipsychotics, antipsychotic polytherapy, and/or high doses. However, little is known about non-antipsychotic psychotropic prescription, or psychotropic medication burden as a more encompassing approach for people treated involuntarily. The aim of this study was to examine the relationship between Mental Health Act (MHA) status and psychotropic polypharmacy and/or high-dose medication prescribing practices in an Australian inpatient mental health unit.

**Methods:**

A retrospective cohort study of 800 adults discharged from a large metropolitan Queensland mental health unit was undertaken. Data was collected for 200 individuals, discharged on at least one psychotropic medicine, at four time periods; Cohort 1 (on or before 31st January 2014), Cohort 2 (2015), Cohort 3 (2016) and Cohort 4 (2017). The number of prescribed medicines and total daily doses were recorded and reviewed for alignment with current clinical guidelines. Participant demographics and clinical characteristics were compared by individual MHA status using chi-square test for categorical variables and analysis of variance for continuous variables. Associations between MHA status and prescribing practices (psychotropic polypharmacy and/or high-dose prescribing) were assessed using bivariate and multivariate binomial logistic regression models. Age, gender, birth country, year of admission, admissions in previous 12 months, primary diagnosis, ECT/clozapine treatment, and other psychotropic medications were adjusted as covariates.

**Results:**

Regression analysis found that compared to their voluntary counterparts, individuals treated involuntarily were 2.7 times more likely to be prescribed an antipsychotic at discharge, 8.8 times more likely to be prescribed more than one antipsychotic at discharge and 1.65 times more likely to be prescribed high-dose antipsychotic treatment at discharge. The adjusted model also found that they were half as likely to be prescribed an antidepressant at discharge.

**Conclusion:**

Implicit review of justifications for increased psychotropic medication burden (antipsychotic polypharmacy and high-doses) in those treated involuntarily is required to ensure clinical outcomes and overall quality of life are improved in this vulnerable group. Clearly documented medication histories, reconciliation at discharge and directions for medication management after discharge are necessary to ensure quality use of medicines.

## Background

In Australia, the Mental Health Act (MHA) is designed to safeguard the rights of individuals who, due to active mental illness, lack sufficient capacity to make informed decisions regarding appropriate treatment [1]. Each state and/or territory has a unique MHA with legislation varying by the respective jurisdiction [2]. Under the Queensland MHA, where this study was undertaken, an individual with active mental illness may be involuntarily admitted to a mental health facility following confirmation by an authorised doctor that they require treatment for the protection of self and/or others from serious harm when no other care options exist [3].

Mental health treatment is guided by the effective management of active psychiatric symptoms, with a recovery-oriented approach focused on treatment planning that meets the individual patient’s needs and goals; often in collaboration with their family and carers [4]. The 1991 United Nations Principals specify that every individual with a mental illness is entitled to the best mental health treatment, irrespective of their MHA status [5]. Whilst concerns for safe and effective use of medication is a priority for all care providers of individuals with a mental illness, those treated involuntarily under the MHA are a particularly vulnerable group. When a person with a mental illness lacks capacity to consent to treatment, authorised doctors may use the MHA to override their consent and provide what they consider as appropriate treatment, including medication. It is therefore imperative for prescribers and the multidisciplinary team to focus on restoring decision-making capacity, remain cognisant of medication choices and effects, and form partnerships with affected individuals and/or their substitute decision makers, i.e. family, carer or support person. This includes seeking and incorporating opinions on medications, providing appropriate medication information, and always treating individuals with dignity and respect [2, 6].

Studies have often demonstrated that individuals treated involuntarily under MHAs experience a higher illness acuity or severity, and greater complexity, longer hospitalisations, and are more likely to be readmitted compared to those treated voluntarily [7–11]. Poor medication adherence and lower treatment satisfaction have also been reported [8, 12]. Research exploring the use of medication and alignment with clinical practice guidelines for this vulnerable population is limited, however, research does suggest an increased likelihood of experiencing treatment outside of clinical practice recommendations such as polypharmacy (i.e. receiving two or more medicines from the same therapeutic group) [7, 13], and psychotropic medications used in doses higher than recommended [14, 15].

To date, most research has focused on discrete elements of antipsychotic prescribing, particularly injectable antipsychotics, polypharmacy and/or high doses, rather than encompassing psychotropics as a whole category [7, 13–19]. Australian and New Zealand research includes studies that have established an association between involuntary community treatment and increased likelihood of long-acting injectable antipsychotics compared to oral antipsychotics [7, 16, 17]. Additionally, a Queensland study reported that inpatients treated involuntarily were significantly more likely to be discharged on antipsychotic polytherapy, high antipsychotic dose, and antipsychotic polytherapy combined with high dose [15]. However, in contrast to McMillan et al. [15], a smaller study of antipsychotic polypharmacy in schizophrenia/schizoaffective disorder in a metropolitan Western Australian hospital found voluntary MHA status was significantly associated with increased rates of polypharmacy [19].

There are a number of service provider, prescriber and consumer rationales for psychotropic medicines being used outside of clinical practice recommendations (e.g. polypharmacy and high doses) including: lack of/partial treatment response; treatment of concomitant symptoms (e.g. anxiety, agitation, insomnia); anticipation of poor adherence; anticipation of symptom relapse upon dose reduction; reluctance to use clozapine; patient/family request; lack of inpatient beds and pressure to reduce admission duration [20–22]. However, there is a lack of evidence to support the clinical benefit and safety of these medication prescribing practices, with many studies reporting increased risks of adverse effects and interactions, tolerability and complexity issues, poorer health outcomes and overall quality of life [23–27]. Consequently, this treatment regime is not endorsed in current Australian or international clinical practice guidelines [28–30].

Given the absence of research taking a more encompassing approach to the prescribing patterns of medicines in the management of mental illnesses, and the associations with individual characteristics (such as MHA status), the aim of the present study was to: (i) examine the relationship between MHA status and rates of psychotropic polypharmacy and/or high-dose psychotropic prescribing at the point of discharge from an urban Queensland adult inpatient mental health unit, across four time periods between 2014 and 2017, and (ii) identify possible areas for improvement of psychotropic prescribing.

## Methods

### Sample and setting

This four-year cohort study extends our earlier research [15], by reviewing all psychotropic medications prescribed at the point of inpatient discharge, (i.e. not just antipsychotics), and by increasing the number of cohorts included. The study reports the discharge medication for all adults treated voluntarily and involuntarily under the MHA, who were prescribed at least one regular psychotropic medicine (i.e. antipsychotic, antidepressant, benzodiazepine, and/or mood stabiliser) from one large Queensland metropolitan public hospital. With 64 adult inpatient psychiatric beds for both voluntary and involuntary individuals admitted under the relevant legislation [3], the hospital is the largest of three adult psychiatric hospitals in the area. The hospital has a catchment area of approximately one million people and encompasses a large culturally and linguistically diverse population.

Data were collected for four cohorts (of 200 patients each who were prescribed at least one regular psychotropic medicine) across four time periods; Cohort 1 (discharged on or before 31st January 2014), Cohort 2 (discharged on or before 31st January 2015), Cohort 3 (discharged on or before 31st January 2016) and Cohort 4 (discharged on or before 31st January 2017). The cohort was identified from an electronic discharge report; the researchers checked the clinical record of each patient to ensure psychotropic medication(s) were prescribed at the point of discharge, moving back in time (from the 31st January of each study year) until a sample of 200 consecutive discharges was achieved. The sample size (*n* = 800) was deemed large enough to provide sufficient statistical power [31], as well as an accurate account of prescribing practices within the selected facility.

Ethical approval was obtained from a University (HSV/04/15/HREC) and District Health Board (HREC/14/QPAH/465) Human Research Ethics Committee.

### Data collection process

Three trained researchers (undertaking research training as part of their Master of Pharmacy degree) collected data from electronic clinical records as per a data collection guide to ensure that a standardised process was followed. The guide provided comprehensive instruction on navigating key hospital-based electronic programs to obtain demographic information (gender, age, country of birth), clinical data (number of prior admissions, primary diagnosis etc) and details of psychotropic medicines (antipsychotic, antidepressant, benzodiazepine, and/or mood stabiliser) prescribed (type, administration route, dose and future follow-up instructions) at the point of discharge. Data accuracy was confirmed by a psychiatric registrar who quality checked a 10% random sample of the total cohort.

Determination of high-dose for antipsychotics, antidepressants and benzodiazepines was calculated by the sum of the total daily dose (TDD) prescribed for each individual divided by the recommended maximum daily dose (MDD); as recommended by therapeutic guidelines (referred to as the total daily equivalent dose).^1^ A score > 1 was classified as high-dose. Therapeutic guidelines used for reference included the Maudsley Prescribing Guidelines in Psychiatry [32] and the British National Formulary [33]. When more than one psychotropic medication within a group was prescribed (e.g. two antipsychotics), a cumulative total was calculated by summing the individual TDD/MDD scores (referred to as the cumulative total daily equivalent dose). Further MDD detail for psychotropic medications, and an example of the calculation method, is provided in Supplementary Tables 1 and 2.

Polypharmacy was determined by the use of two or more medicines from the same group simultaneously (e.g. antipsychotics), or use of more than one formulation of the same medicine.

MHA status at the point of discharge, irrespective of status on admission, was collected and categorised as either voluntary or involuntary.

### Statistical analyses

Data analyses were conducted using Stata version 13.1 (StataCorp LP, USA). Participant demographic and clinical characteristics were compared by individual MHA status using chi-square test for categorical variables and analysis of variance for continuous variables. Associations between MHA status and psychotropic polypharmacy and/or high-dose prescribing were assessed using bivariate and multivariate binomial logistic regression models. Four multivariate logistic regression models were employed. To investigate MHA status and polypharmacy treatment, all four psychotropic groups were included in Model 1. Other covariates including age, gender, birth country, year of admission, admission duration, admissions in previous 12 months, primary diagnosis, ECT/clozapine treatment, were adjusted for. To examine associations between MHA status and high-dose prescribing for the three psychotropic medication groups (antipsychotics, antidepressants and benzodiazepines), 647 individuals prescribed one or more antipsychotics were included in Model 2, 327 individuals prescribed one or more antidepressants were included in Model 3, and 221 individuals prescribed one or more benzodiazepines were included in Model 4. Apart from the above covariates, the total number of psychotropic medications prescribed at discharge was adjusted for, in Models 2–4. Statistical significance was declared at *p* < 0.05.

## Results

Table 1 outlines the demographic and clinical characteristics for the total study population (*n* = 800); just over half were male (57.4%), three-quarters were born in Australia (75.4%), and the mean age was 38.5 years (SD = 12.0 years; range = 18–69 years). The median length of hospital admission was nine days (IQR = 12 days; range = 1–384 days) and most of the study population (73.8%) had a previous admission recorded in the 12 months prior to the episode included in this study. The most common primary discharge diagnosis was schizophrenia (32.4%) and just over a third of the study population had a substance use disorder recorded at discharge (36.8%). Of the total population, 36.6% were discharged receiving involuntary treatment under the MHA. Table 1 outlines the differences between the two groups at discharge (voluntary vs involuntary treatment) which included country of birth, cohort, length of admission, number of admissions in last 12 months, diagnosis, ECT and previous clozapine treatment.

**Table 1:** Participant characteristics (*n* = 800)

	MHA status		p
	Voluntary n (%)	Involuntary n (%)	
**Total cohort population**	**507 (63.4)**	**293 (36.6)**	
Gender (male)	284 (56.0)	175 (59.7)	0.3
Age (years), mean (SD)	38.1 (12.1)	38.8 (11.9)	0.6
Country of birth			0.03
Australia	397 (78.3)	206 (70.3)	
Asia	45 (8.9)	26 (8.9)	
Pacific^a^	25 (4.9)	23 (7.8)	
Other (Africa, Americas, Europe)	40 (7.9)	38 (13.0)	
Cohort			< 0.001
1	155 (77.5)	45 (22.5)	
2	130 (65.0)	70 (35.0)	
3	128 (64.0)	72 (36.0)	
4	94 (47.0)	106 (53.0)	
Length of admission (days), median (IQR)	7 (10)	15 (16)	< 0.001
Admissions (last 12 months)			0.003
None	122 (24.0)	88 (30.0)	
One	229 (45.2)	93 (31.7)	
Two	83 (16.4)	58 (19.8)	
≥ Three	73 (14.4)	54 (18.5)	
Primary diagnosis			< 0.001
Schizophrenia	102 (20.1)	157 (53.6)	
Bipolar disorder	41 (8.1)	52 (17.8)	
Other psychotic disorder	40 (7.9)	27 (9.2)	
Other mood disorder	174 (34.3)	25 (8.5)	
Other^b^	150 (29.6)	32 (10.9)	
Substance use disorder	189 (37.3)	105 (35.8)	0.7
ECT treatment during admission	9 (1.8)	26 (8.9)	< 0.001
Previous/current clozapine treatment	24 (4.7)	37 (12.6)	< 0.001

*SD* Standard Deviation, *IQR* Interquartile Range; ^a^Pacific included New Zealand, Samoa, Tonga, Papua New Guinea; ^b^Other diagnoses included personality disorder, stress reaction, no major mental illness, cognitive/intellectual impairment, alcohol/substance use/abuse

### Psychotropic polytherapy at discharge

The average number of psychotropic medications prescribed at discharge was two (range = 1–6). Table 2 outlines the prescribing rates of each of the four psychotropic groups; the majority (80.9%) of the cohort were prescribed one or more antipsychotics at discharge; 40.9% were prescribed one or more antidepressants; slightly more than a quarter were prescribed one or more benzodiazepines (27.6%); and a quarter were prescribed one or more mood stabilisers (25.1%). Of the total cohort population (*n* = 800), only 13 individuals (1.6%) were concomitantly prescribed one or more medicines from each of the four psychotropic groups. The highest number of psychotropic medications prescribed at discharge was six which occurred for two individuals (Table 2).

**Table 2:** Psychotropic medication groups prescribed on discharge (*n* = 800)

Number of medications prescribed per individual	n	%
**Psychotropic group combinations**		
One psychotropic group	356	44.5
Two psychotropic groups	305	38.1
Three psychotropic groups	126	15.8
All four psychotropic groups	13	1.6
**Total number of psychotropic medications prescribed**		
One	251	31.4
Two	293	36.6
Three	184	23.0
Four	57	7.1
Five	13	1.6
Six	2	0.3
**Antipsychotic**		
None	153	19.1
One	428	53.5
≥ Two	219	27.4
**Mood stabiliser**		
None	599	74.9
One	174	21.8
≥ Two	27	3.4
**Antidepressant**		
None	473	59.1
One	296	37.0
≥ Two	31	3.9
**Benzodiazepine**		
None	579	72.4
One	213	26.6
Two	8	1.0

Table 3 shows the prescribing rates of the most commonly prescribed psychotropic medications at discharge. Overall olanzapine was the most commonly prescribed antipsychotic (26.7%). In terms of long-acting injectable (LAI) antipsychotics, paliperidone accounted for 48.4% of all LAIs prescribed. Antipsychotic polypharmacy occurred in 27.4% of the study population (*n* = 208 prescribed two and *n* = 11 prescribed three) and most commonly this consisted of co-prescribed oral and LAI formulations (*n* = 177).

**Table 3:** Prescription rates of medications most frequently prescribed and high-dose rates on discharge (*n* = 800)

Medication	n (%)	Route		High-dose
		Oral n	LAI n	(TDD/MDD > 1)
**Antipsychotics**	**877**	**658**	**219**	**75 (8.6)**
Olanzapine	234 (26.7)	223	11	41
Risperidone	188 (21.4)	161	27	10
Quetiapine	140 (16.0)	140	nil	10
Paliperidone	107 (12.2)	1	106	12
Aripiprazole	52 (5.9)	39	13	1
Zuclopenthixol	43 (4.9)	3	40	nil
Clozapine	42 (4.8)	42	nil	nil
Other	71 (8.1)	49	22	1
**Antidepressants**	**359**	**N/A**	**N/A**	**26 (7.2)**
Mirtazapine	74 (20.6)			12
Venlafaxine	58 (16.2)			1
Sertraline	40 (11.1)			1
Fluoxetine	38 (10.6)			1
Duloxetine	34 (9.5)			nil
Other	115 (32.0)			11
**Benzodiazepines**	**229**	**N/A**	**N/A**	**6 (2.6)**
Diazepam	167 (72.9)			1
Lorazepam	29 (12.7)			3
Temazepam	18 (7.9)			nil
Other	15 (6.6)			2
**Mood stabilisers**	**229**	**N/A**	**N/A**	**N/A**^a^
Sodium valproate	117 (51.1)			
Lithium	97 (42.4)			
Other	15 (6.6)			

^a^High dose information not available for mood stabilisers. *LAI* long-acting injectable antipsychotic, *TDD* total daily dose, *MDD* maximum daily dose

Mirtazapine was the most commonly prescribed antidepressant (20.6%), followed by venlafaxine (16.2%). Most of those treated with antidepressants were only prescribed one agent ; antidepressant polypharmacy occurred in just 3.9% of the study population (*n* = 31/800).

Sodium valproate was the most commonly prescribed mood stabiliser (*n* = 117/229;51.1%), followed by lithium (*n* = 97/229;42.4%). Mood stabiliser polypharmacy only occurred in 3.4% of the study population (*n* = 27/800).

The most commonly prescribed benzodiazepine was diazepam (72.9%), followed by lorazepam (12.7%). Only eight individuals (1.0% of study population) were prescribed two benzodiazepines concurrently.

### High-dose prescribing at discharge

Overall a quarter of the total study population (*n* = 200/800) were prescribed a cumulative dose of psychotropic medication considered to be high-dose (i.e. a cumulative total daily equivalent dose > 1). This included 158 individuals prescribed high-dose antipsychotic treatment (24.4% of those prescribed antipsychotics), 45 individuals prescribed high-dose antidepressant treatment (13.8% of those prescribed antidepressants) and six people prescribed high-dose benzodiazepine treatment (2.7% of those prescribed benzodiazepines). Eight individuals were prescribed high-dose treatment from more than one psychotropic group; seven prescribed high-dose treatment from two groups and one prescribed high-dose treatment from all three groups).

In total, 8.6% (*n* = 75/877) of antipsychotic prescriptions were identified as high-dose (i.e. a total daily equivalent antipsychotic dose > 1), most commonly with olanzapine prescriptions; 7.2% (*n* = 26/359) of antidepressant prescriptions were identified as high-dose, most commonly with mirtazapine prescriptions; and 2.6% (*n* = 6/229) of benzodiazepine prescriptions were high-dose, most commonly with lorazepam prescriptions (Table 3).

### Multivariate logistic regression

In Model 1 (Table 4) associations between MHA status and the number of psychotropic medicines for each of the four therapeutic groups (antipsychotic, antidepressant, mood stabiliser and benzodiazepine) at discharge were explored. Results show that after adjustment three significant associations were found with MHA status: being prescribed one, or more than one antipsychotic and being prescribed an antidepressant. Those treated involuntarily were 2.7 times more likely to be prescribed an antipsychotic at discharge, 8.8 times more likely to be prescribed more than one antipsychotic at discharge and half as likely to be prescribed an antidepressant at discharge compared to those treated voluntarily.

**Table 4:** Bivariate and multivariate logistic regression models (associations between MHA status and psychotropic prescribing on discharge)

**Model 1**^**a**^*n* = 800				
	**MHA Status**		**Crude Odds Ratio**	**Adjusted Odds Ratio**
**Variable**	**Voluntary**	**Involuntary**	**(95% CI)**	**(95% CI)**
Antipsychotic	n (%)	n (%)		
None	144 (28.4)	9 (3.1)	Reference	Reference
One	291 (57.4)	137 (46.7)	7.53 (3.73,15.22)**	2.71 (1.22, 6.04)*
> One	72 (14.2)	147 (50.2)	32.7 (15.7, 67.8)**	8.80 (3.69, 20.98)**
Antidepressant	n (%)	n (%)		
None	237 (46.8)	236 (80.5)	Reference	Reference
One	243 (47.9)	53 (18.1)	0.22 (0.15, 0.31)**	0.47 (0.30, 0.74)*
> One	27 (5.3)	4 (1.4)	0.15 (0.05, 0.43)**	0.31 (0.08, 1.19)
Mood stabiliser	n (%)	n (%)		
None	407 (80.3)	192 (65.5)	Reference	Reference
One	90 (17.7)	84 (28.7)	1.98 (1.40, 2.79)**	1.35 (0.84, 2.17)
> One	10 (2.0)	17 (5.8)	3.60 (1.62, 8.02)*	2.17 (0.80, 5.89)
Benzodiazepine	n (%)	n (%)		
None	354 (69.8)	225 (76.8)	Reference	Reference
One/Two	153 (30.2)	68 (23.2)	0.70 (0.50, 0.97)*	1.00 (0.66, 1.53)
**Models 2, 3 and 4**^**b**^				
	**MHA Status**		**Crude Odds Ratio**	**Adjusted Odds Ratio**
**Variable**	**Voluntary**	**Involuntary**	**(95% CI)**	**(95% CI)**
Model 2 *n* = 647	n (%)	n (%)		
Cumulative antipsychotic equivalent total dose > 1	56 (15.4)	102 (35.9)	3.07 (2.11, 4.47)**	1.65 (1.05, 2.59)*
Model 3 *n* = 327	n (%)	n (%)		
Cumulative antidepressant equivalent total dose > 1	36 (13.3)	9 (15.8)	1.22 (0.55, 2.70)	1.05 (0.37, 2.97)
Model 4 *n* = 221	n (%)	n (%)		
Cumulative benzodiazepine equivalent total dose > 1	4 (2.6)	2 (2.9)	1.13 (0.20, 6.32)	0.92 (0.12,6.74)

*p < 0.05, **p < 0.001

*CI* confidence interval, *ECT* Electroconvulsive therapy

^a^Adjusted for age, gender, country of birth, year of admission length of admission, admissions in last 12 months, primary diagnosis, ECT during admission, previous clozapine treatment, and three other psychotropic medicines groups on discharge;

^b^Adjusted for age, gender, country of birth, year of admission, length of admission, admissions in last 12 months, primary diagnosis, ECT during admission, previous clozapine treatment, and total number of psychotropic medicines on discharge;

In Models 2, 3 and 4 (Table 4) associations between MHA status and high-dose treatment (cumulative total daily equivalent dose > 1) with (i) antipsychotics, (ii) antidepressants, or (iii) benzodiazepines were explored. The models were adjusted for age, gender, country of birth, cohort, length of admission, admissions in the last 12 months, primary diagnosis, ECT treatment during admission, previous clozapine treatment, and the total number of psychotropic medicines prescribed on discharge. Table 4 shows that after adjustment only one significant association was found with MHA status: being prescribed high-dose antipsychotic treatment. Those treated involuntarily were 1.65 times more likely to be prescribed high-dose antipsychotic treatment at discharge compared to those people treated voluntarily.

## Discussion

This study was novel in that we comprehensively explored (i) prescribing across psychotropic medications rather than solely focusing on antipsychotic treatment, and (ii) psychotropic medication burden in terms of polypharmacy (with respect to four psychotropic groups; antipsychotics, antidepressants, mood stabilisers and benzodiazepines) and high-dose treatment (with respect to three psychotropic groups; antipsychotics, antidepressants and benzodiazepines).

In summary, this study found that the prescribing pattern of psychotropic medication, specifically antipsychotics, aligns with other Australian and international research [7, 13–18]. That is, individuals treated involuntarily under the MHA at discharge were significantly more likely to be prescribed antipsychotic treatment, antipsychotic polypharmacy and/or high-dose antipsychotic treatment at discharge. However, in this study we also found that individuals treated involuntarily under the MHA were less likely to be prescribed antidepressant treatment at discharge when compared to those treated voluntarily, and this remained significant even when the model was adjusted for primary diagnosis. Overall there were high rates of antipsychotic prescribing outside of practice recommendations in the total cohort with 27% of individuals prescribed polypharmacy and 24% prescribed high-dose treatment. In contrast, low rates of prescribing outside of practice recommendations were found for other psychotropic medications: polypharmacy was found in less than 4% of individuals prescribed antidepressants and mood stabilisers and only 1% for benzodiazepines; and high-dose treatment was found in less than 14% of those prescribed antidepressants and less than 3% of those prescribed benzodiazepines.

Whilst there are a number of studies exploring antipsychotic use and adherence to prescribing guidelines for people with mental illness in inpatient, community and prison populations, we were only able to find two studies reporting psychotropic use more comprehensively in a population diagnosed with a mental illness [18, 19]. Gisev et al. [18] described the use of psychotropic medication in a group of 378 people treated involuntarily in a community setting in New South Wales (Australia): all but one were prescribed antipsychotic treatment (32% polypharmacy and up to 27% high-dose antipsychotics), 24% were prescribed a mood stabiliser, 9.9% an antidepressant, and 2.4% a benzodiazepine. Similarly, a Western Australian study [19], described psychotropic use in a group of 243 people admitted to a mental health unit with a diagnosis of schizophrenia: 94% were prescribed antipsychotic treatment (43% polypharmacy), 26% were prescribed a mood stabiliser, 21% an antidepressant, and 22% a benzodiazepine. Neither of these studies explored polypharmacy/and or high dose prescribing more widely than antipsychotics. To our knowledge, our study is the first study exploring prescribing practices more comprehensively.

In this four-year cohort study individuals treated involuntarily were almost three times more likely to be prescribed antipsychotic treatment at discharge, almost nine times more likely to be discharged on more than one antipsychotic, and about one and a half times more likely to be prescribed high-dose antipsychotic treatment when compared to those treated voluntarily. Our findings regarding increased odds for antipsychotic polypharmacy and high-doses with involuntary treatment are consistent with those of a 1998 multi-centre census of 3576 inpatients prescribed antipsychotics in the United Kingdom [13]. A 2012 audit of 272 inpatients in Portugal, however, found that whilst involuntary treatment was a significant predictor of antipsychotic high-doses this was not the case for polypharmacy [14].

According to a 2012 systematic review, rates of antipsychotic polypharmacy prescribing vary considerably in the literature ranging from 6 to 90%, with a median global prevalence of 19.6% [34]. Given the lack of evidence, such prescribing remains controversial and outside of clinical guidelines. Whilst the observed rate in this study is at the lower end of the range, it is higher than the reported global prevalence; more than a quarter of the individuals in our study (27.4%) were exposed to the risks associated with multiple antipsychotics, including higher doses, and potentially a greater number of adverse effects. This study provides a timely reminder that providing regular education about appropriate indications for psychotropic medications alongside realistic timepoints for reviewing medications to ensure that they are appropriate or of continued benefit, is vital to ensure the quality use of psychotropics [35]. A study by Tyson et al. [36], suggested that once more than one antipsychotic is prescribed it is difficult for prescribers to keep track of the total antipsychotic dose. This is particularly important for those people treated involuntarily under the MHA, who were more likely to be prescribed more than one antipsychotic and at higher doses.

Prescribed treatment regimens of polypharmacy and high-doses at discharge may not necessarily have reflected the ongoing therapeutic intentions of the prescriber. For example, the prescriber may have intended these medicines to be used for short periods while in the process of switching antipsychotics or down titrating a dose as the acute episode resolved. However, in these situations it is imperative that the prescriber communicates treatment recommendations, via the discharge summary, to the team who are providing care after discharge, including the general practitioner and/or community mental health team, and with the patient and their family members/carer at the point of planning discharge. Additionally, if polypharmacy and/or high-dose treatment was the prescribers intention, then it is important that the rationale and justification is provided, together with a monitoring plan which is clearly documented in the clinical records and similarly communicated to the discharge care providers. Unfortunately it was beyond the scope of this study to explore the clinical notes and discharge summaries for evidence of treatment justifications and communication methods for the hand-over of such information, or to review prescribing regimens for those individuals with antipsychotic prescribing outside of practice recommendations once they had returned to the community setting. Future research could focus on this gap in the literature.

While education is an ongoing and necessary intervention to encourage rational prescribing practices, other avenues could also be explored to improve prescribing practices for this vulnerable population. For example, the role and contribution of mental health pharmacists as part of the multidisciplinary team to improve the quality use of psychotropic medications in psychiatric settings is increasingly recognised [37, 38]. In 2019, the service setting for this study increased the Full Time Equivalent of mental health pharmacists from 3.0 to 4.5; the first real increase in more than 15 years. The team provides dedicated mental health pharmacy services to acute inpatient and community mental health settings, including adult, older persons and clozapine clinics. They regularly review psychotropic medication regimens; complete medicines reconciliation at admission and discharge; solve medication-related problems, document medication histories, and contribute to multidisciplinary team discussions including discharge planning. Exploring the impact of these expanded medication-related services, alongside electronic prescribing (implemented service wide in March 2017) and the recently introduced Queensland MHA (2016), on psychotropic prescribing for this involuntary treatment population will be an important focus of future research.

Finally, the increase in involuntary treatment rates in the final cohort in this study (53% Cohort 4 vs. 22.5% Cohort 1), is worthy of comment. Whilst this study was not designed to explore MHA rates over time or of decision-making regarding involuntary treatment, this trend is interpreted in the context of the challenges facing mental health services in Australia [39, 40]. Nationwide there has been an increase in demand for acute adult psychiatric beds, with concerns expressed that Australia lacks the required inpatient resources [41]. With increasing pressure on these resources, the threshold or acuity for an admission may increase, resulting in higher rates of involuntary admissions. Consequently, the findings and recommendations of this study are of growing importance.

### Strengths and limitations

This study encompassed a large cohort (*n* = 800) across four time periods. Rather than focusing solely on antipsychotics the study provided a holistic insight into psychotropic polypharmacy and high-dose prescribing rates. This is a previously unreported area in the international literature. Hence, the results increase our understanding of the relationship between psychotropic polypharmacy and high-dose prescribing practices and will contribute towards the continuous update of local, national, and international policy and mental health clinical guidelines.

The results of this retrospective cohort study must be considered with respect to its limitations. The data extracted were dependent on the availability of routinely collected data points and the accuracy of clinical data documented by clinicians, which can often lack completeness and reasoning behind clinical judgement and decision-making. For example it was not possible to collect data on severity as it is not routinely collected, and whilst we adjusted for covariates such as diagnosis, admission duration, ECT/clozapine treatment we were not able to adjust for severity of illness which may have impacted on treatment at discharge. Further, this study was an explicit review of prescribing patterns and did not explore the implicit reasoning for the use of prescribing outside of clinical practice recommendations; therefore rates of polypharmacy may have been over-estimated. For example, there may have been situations when antipsychotic polypharmacy may have been valid (i.e. for cross-titration purposes). The use of dose equivalencies for medication comparisons within a class is useful, but potency equivalencies do not consider pharmacokinetic differences between them. Furthermore, side-effect profiles may vary in a way that is not well portrayed by the daily dose potency equivalence calculations [42]. Finally, as this study was undertaken in only one Australian clinical setting, the results may not be generalisable to other clinical settings.

## Conclusion

Given the treatment burden and potential complications that may result from antipsychotic polypharmacy and/or high-dose prescribing, this research highlights the importance of a regular prescribing review, ongoing education, and the establishment of treatment guidelines for this vulnerable group. It is important that mental health professionals (including psychiatrists, registrars, pharmacists, nurses and allied health staff) acknowledge that an individual’s treatment status under the MHA is not only a legal issue, but rather signifies an important characteristic associated with increased clinical risk and vulnerability. Consideration and attentiveness to these potential risks also underpins the core values of the Queensland Mental Health Act (2016) and the need to use the least restrictive means possible when using involuntary treatment. We were also unable to locate a current Royal Australian and New Zealand College of Psychiatrists (RANZCP) position statement or guideline with specific respect to psychotropic polypharmacy, the creation of which would be recommended by this research.

This cohort study also highlighted the sparse research focused on non-antipsychotic psychotropic prescription, and the importance of considering psychotropic medication burden holistically for people with a severe and persistent mental illness. This is especially so for those subject to involuntary treatment under a MHA who lack sufficient capacity to make informed decisions regarding what others consider to be necessary treatment.

## Supplementary information

**Additional file 1: Table S1.** Maximum daily doses (MDD) of psychotropic medications as per clinical guidelines [31, 32].

**Additional file 2: Table S2.** Example calculation methods for total daily equivalent dose (TDD) (per 24 h) of psychotropic medications (McMillan et al., 2017).

## Data Availability

The data that support the findings of this study are available from the study site but restrictions apply to the availability of these data, which are not publicly available.

## Abbreviations

LAI: Long-acting injectable
MHA: Mental Health Act
RANZCP: Royal Australian and New Zealand College of Psychiatrists
TDD: Total daily dose
MDD: Maximum daily dose

## Footnotes

1 High-doses were not calculated for mood stabilisers due to lack of standardisation in maximum therapeutic dose expression (Supplementary Table 1).
