# Supplementary material for: Is higher psychotropic medication burden associated with involuntary treatment under the Mental Health Act? A four-year Australian cohort study
Source: BMC Psychiatry. 2020 Jun 11;20:294. doi: 10.1186/s12888-020-02661-6 (PMC7291489; doi:10.1186/s12888-020-02661-6)
Supplement: Supplementary file 1 — Additional file 1: Table S1. Maximum daily doses (MDD) of psychotropic medications as per clinical guidelines [31, 32]. [file 12888_2020_2661_MOESM1_ESM.docx]

**Supplementary Table 1:** Maximum daily doses (MDD) of psychotropic medications as per clinical guidelines [31, 32]

| **Medication** | **Maximum therapeutic dose** | **Medication** | **Maximum therapeutic dose** |
| --- | --- | --- | --- |
| **Antipsychotics** | | **Antidepressants** | |
| Amisulpride | 1200mg PO daily | Agomelatine | 50mg PO daily |
| Aripiprazole | 400mg LAI every 4 weeks | Amitriptyline | 200mg PO daily |
|  | 30mg PO daily | Citalopram | 60mg PO daily |
| Asenapine | 20mg PO daily | Clomipramine | 250mg PO daily |
| Chlorpromazine | 1000mg PO daily | Desvenlafaxine | 200mg PO daily |
| Clozapine | 900mg PO daily | Doxepin | 300mg PO daily |
| Droperidol | 20mg IM/IV daily | Duloxetine | 120mg PO daily |
| Flupenthixol | 18mg PO daily | Escitalopram | 20mg PO daily |
|  | 400mg LAI every week | Fluoxetine | 60mg PO daily |
| Fluphenazine | 100mg LAI every 2 weeks | Fluvoxamine | 300mg PO daily |
| Haloperidol | 30mg PO daily | Imipramine | 300mg PO daily |
|  | 300mg LAI every 4 weeks | Mianserin | 90mg PO daily |
| Lurasidone | 160mg PO daily | Mirtazapine | 45mg PO daily |
| Olanzapine | 30mg PO daily | Nortriptyline | 150mg PO daily |
|  | 300mg LAI every 4 weeks | Paroxetine | 60mg PO daily |
| Paliperidone | 12mg PO daily | Reboxetine | 12mg PO daily |
|  | 150mg LAI every 4 weeks | Sertraline | 200mg PO daily |
| Periciazine | 300mg PO daily | Venlafaxine | 375mg PO daily |
| Quetiapine | 800mg PO daily | Vortioxetine | 20mg PO daily |
| Risperidone | 16mg PO daily |  | |
|  | 50mg LAI every 2 weeks |  |  |
| Ziprasidone | 160mg PO daily |  |  |
| Zuclopenthixol | 150mg PO daily |  |  |
|  | 600mg LAI every week |  |  |
| **BELOW SECTION NEEDS FIXING – all items need to be in a single cell ie as above** | | | |
| **Mood Stabilisers** | | **Benzodiazepines** |  |
| Lithium | Highest tolerated dose without significant adverse effect | Alprazolam | 3mg PO daily |
| Sodium Valproate | 60mg/kg PO daily | Clonazepam | 8mg PO daily |
| Carbamazepine | 1600mg PO daily | Diazepam | 60mg PO daily |
| Oxcarbazepine | 1200mg PO daily | Lorazepam | 4mg PO daily |
| Lamotrigine | 400mg PO daily | Nitrazepam | 10mg PO daily |
| Topiramate | 500mg PO daily | Oxazepam | 120mg PO daily |
|  |  | Temazepam | 40mg PO daily |
