# Supplementary material for: Is higher psychotropic medication burden associated with involuntary treatment under the Mental Health Act? A four-year Australian cohort study
Source: BMC Psychiatry. 2020 Jun 11;20:294. doi: 10.1186/s12888-020-02661-6 (PMC7291489; doi:10.1186/s12888-020-02661-6)
Supplement: Supplementary file 2 — Additional file 2: Table S2. Example calculation methods for total daily equivalent dose (TDD) (per 24 h) of psychotropic medications (McMillan et al., 2017). [file 12888_2020_2661_MOESM2_ESM.docx]

**Supplementary Table 2:** Example calculation methods for total daily equivalent dose (TDD) (per 24 hours) of psychotropic medications (McMillan et al., 2017)

| **Medication Class** | **Total Daily Dose (per 24hours) Calculation Method** |
| --- | --- |
| **Antipsychotics**  **Antidepressants**  **Benzodiazepines** | Dose = Total Daily Dose  Maximum Daily Dose (Supplementary Table 1)  Where a score > 1 is considered a ‘High-dose’  (i.e)  Paliperidone 3mg a day plus 150mg LAI every 4 weeks  Dose = 3mg (TDD) plus Dose = 150mg (TDD)  12mg (MDD) 150mg (MDD)  = 0.25 + 1.0  = 1. 25 > 1, therefore a “High-dose” |
